# Editorial: Rehabilitation to guide functional plasticity and regeneration with novel cellular, pharmacological and neuromodulation therapies

**DOI:** 10.3389/fresc.2025.1563975

**Published:** 2025-02-13

**Authors:** Régis Gemerasca Mestriner, Sukhvinder Kalsi-Ryan, Gita Gholamrezaei, Gustavo Balbinot

**Affiliations:** ^1^ Biomedical Gerontology Program of the School of Medicine, Pontifical Catholic University of Rio Grande do Sul (PUCRS), Porto Alegre, Brazil; ^2^ Neurosciences, Motor Behavior and Rehabilitation Research Group (NECORE), PUCRS, Porto Alegre, Brazil; ^3^ School of Health and Life Sciences, Pontifical Catholic University of Rio Grande do Sul, Porto Alegre, Brazil; ^4^ Geriatric and Gerontology Institute, PUCRS, Porto Alegre, Brazil; ^5^ KITE Research Institute|Toronto Rehab, University Health Network, Toronto, ON, Canada; ^6^ Department of Biomedical Physiology and Kinesiology, Simon Fraser University, Burnaby, BC, Canada; ^7^ Movement Neurorehabilitation and Neurorepair Laboratory, Simon Fraser University, Burnaby, BC, Canada; ^8^ Institute for Neuroscience and Neurotechnology, Simon Fraser University, Burnaby, BC, Canada

**Keywords:** spinal cord injuries, stroke, cerebral palsy, neuromodulation, rehabilitation

**Editorial on the research topic**
Rehabilitation to guide functional plasticity and regeneration with novel cellular, pharmacological and neuromodulation therapies

While we, as a research field, strive to improve outcomes for people with neurological conditions, we understand that no single therapy or intervention can work in isolation. Combining methods represents the future of optimizing outcomes in rehabilitation. Research on combinatorial treatments remains limited. While some studies have explored the combination of exercise- or activity-based therapies with neuromodulation, little has been done to investigate the integration of neuromodulation with cellular or pharmacologic treatments. Given the etablished safety of a broad range of neuromodulation techniques, there is an interesting opportunity to further investigate the potential benefits of combining pharmacologic approaches with neuromodulation.

The quest to restore function following neurological injuries continues to drive innovation in the field of rehabilitation. Despite the complexity of central nervous system injuries and the limited capacity for regeneration, promising avenues are emerging. By integrating rehabilitation with cutting-edge cellular therapies, pharmacological interventions, and neuromodulation strategies, researchers aim to harness the body's inherent plasticity to facilitate recovery and functional regeneration.

Spinal cord injury (SCI) rehabilitation offers a compelling example of these advancements. Cervical SCI disrupts critical neural circuits controlling upper limb function. While endogenous repair mechanisms promote reorganization and adaptive plasticity in sparred circuits, maladaptive rewiring can hinder functional recovery (1–6). Therefore, strategies targeting the functional rewiring of motor pathways are essential to enhance meaningful recovery. Multiple preclinical (7, 8) and clinical (9, 10) studies have demonstrated that rehabilitation improves functional recovery after SCI by training the spared motor networks and providing activity-dependent feedback to locomotor pathways. For instance, Gregoire Courtine's research on neuromodulation for SCI recovery in humans highlights the integration of rehabilitation strategies with epidural (11) or transcutaneous spinal cord stimulation (12), brain–spine interfaces (13, 14), and hypothalamic deep brain stimulation (15). Importantly, the neuroplastic changes induced by rehabilitation training are dependent on the type of adopted training paradigm (16). Strength training primarily modulates motor network excitability and increases number of synapses, whereas skilled motor training elicits broader mechanisms, including synapse formation, enhanced synaptic strength, and network reorganization (16). In stroke, studies on anti-NOGO therapy demonstrate that its efficacy is optimized when combined sequentially with an appropriate rehabilitation regimen (17). These examples underscore the critical need for combined and targeted rehabilitation paradigms.

Building on these concepts, this research topic examines perspectives on combining rehabilitation with advanced therapies, including stem cell applications for SCI (Balbinot), the safety of Hebbian-type stimulation (Haakana et al.), personalized strategies for pediatric cerebral palsy (Behboodi et al., Raess et al.), and the sex-specific effects of acrobatic training on cognitive decline induced by cerebral hypoperfusion (Martini et al.).

Balbinot emphasizes the synergy between targeted rehabilitation and stem cell-based therapies, particularly for improving upper extremity function in cervical SCI. Preclinical studies highlight the necessity of combining regenerative strategies with rehabilitation protocols that mirror clinical practices, notably using neuromodulation to activate neural circuits below the injury level. Techniques such as corticospinal tract and spinal cord stimulation represent a promising frontier to enhance the efficacy of cell-based therapies for severe upper extremity paralysis. The convergence of these approaches holds significant hope for unlocking new treatments in the clinical setting.

Adding further depth, a novel neuromodulation protocol of paired associative stimulation (high PAS), combines high-intensity transcranial magnetic stimulation with high-frequency peripheral nerve stimulation to target corticospinal tract plasticity (Haakana et al., 18, 19). Preliminary findings by Haakana et al. on heart rate variability indicate that this approach is safe, inducing short-term modulation of parasympathetic activity without sustained cardiovascular effects. High PAS has the potential to enhance rehabilitation for neurological conditions, further emphasizing the need for continued exploration of its systemic impacts—especially when combined with other plasticity enhancing approaches.

In parallel, the adaptability and therapeutic potential of neurological interventions extend to pediatric conditions such as cerebral palsy. Functional electrical stimulation has demonstrated mixed results in improving gait kinematics (Behboodi et al.). This highlights the importance of identifying neurotherapeutic responders to optimize individualized protocols tailored to individual needs. Furthermore, combining robotic rehabilitation paired with transcranial direct current stimulation shows promise for enhancing upper extremity motor outcomes. Raess et al. show that, despite logistical challenges and patient-specific barriers, this combination shows feasibility and tolerability, providing a foundation for future research to elucidate optimal protocols for clinical application.

Finally the challenge of treating chronic cerebral hypoperfusion is addressed through innovative strategies such as acrobatic training (Martini et al.). Martini et al. show that this intervention mitigates astrocytic remodeling in hippocampal subfields associated with spatial memory impairments while uncovering sex-specific response. In males, training appears to increase astrocyte populations and improve memory retention, whereas in females, it enhances cell viability, highlighting the nuanced interplay between rehabilitation therapies and sex-specific cellular plasticity.

In conclusion, the integration of advanced cellular, pharmacological, and neuromodulation therapies with comprehensive rehabilitation strategies heralds a new era of possibilities for functional recovery in neurological conditions. While challenges remain, it is imperative to rigorously assess the biological plausibility of these technologies as a cornerstone of their validation. Drawing from the Bradford-Hill criteria (20), this focus on plausibility ensures that the mechanisms driving neural regeneration and plasticity are both scientifically credible and capable of being translated into effective clinical applications. Such a framework is essential for harnessing these interventions to maximize neuroplasticity and advance the field of rehabilitation sciences.
